# Preceramic Paper-Derived SiC_f_/SiC_p_ Composites Obtained by Spark Plasma Sintering: Processing, Microstructure and Mechanical Properties

**DOI:** 10.3390/ma13030607

**Published:** 2020-01-29

**Authors:** Ke Li, Egor Kashkarov, Maxim Syrtanov, Elizaveta Sedanova, Alexander Ivashutenko, Andrey Lider, Ping Fan, Daqing Yuan, Nahum Travitzky

**Affiliations:** 1 School of Nuclear Science and Engineering, National Research Tomsk Polytechnic University, 634050 Tomsk, Russia; libeichen@mail.ru (K.L.); maxim-syrtanov@mail.ru (M.S.); eps4@tpu.ru (E.S.); ivaschutenko@mail.ru (A.I.); lider@tpu.ru (A.L.); nahum.travitzky@fau.de (N.T.); 2 China Institute of Atomic Energy, Beijing 102413, China; fanping@ciae.ac.cn (P.F.); yuandq@ciae.ac.cn (D.Y.); 3 Department of Materials Science, Glass and Ceramics, Friedrich-Alexander-Universität Erlangen-Nürnberg, 91054 Erlangen, Germany

**Keywords:** ceramic matrix composites, preceramic paper, silicon carbide, fibers, spark plasma sintering, small punch test, X-ray computed tomography, microstructure, mechanical properties

## Abstract

Ceramic matrix composites (CMCs) based on silicon carbide (SiC) are promising materials for applications as structural components used under high irradiation flux and high temperature conditions. The addition of SiC fibers (SiC_f_) may improve both the physical and mechanical properties of CMCs and lead to an increase in their tolerance to failure. This work describes the fabrication and characterization of novel preceramic paper-derived SiC_f_/SiC_p_ composites fabricated by spark plasma sintering (SPS). The sintering temperature and pressure were 2100 °C and 20–60 MPa, respectively. The content of fibers in the composites was approx. 10 wt.%. The matrix densification and fiber distribution were examined by X-ray computed tomography and scanning electron microscopy. Short processing time avoided the destruction of SiC fibers during SPS. The flexural strength of the fabricated SiC_f_/SiC_p_ composites at room temperature varies between 300 and 430 MPa depending on the processing parameters and microstructure of the fabricated composites. A quasi-ductile fracture behavior of the fabricated composites was observed.

## 1. Introduction

Silicon carbide (SiC) has excellent properties such as low density, high specific strength, high specific modulus, resistance to thermal shock, low activation, radiation tolerance and corrosion resistance [1]. However, like other ceramic materials, high brittleness of SiC has become disadvantage of its application as a structural material [2]. SiC matrix composites reinforced with continuous SiC fibers reduce the macroscopic brittleness of the material and are characterized by quasi-ductile behavior under mechanical loading [3,4]. An interface coating such as BN or pyrolytic carbon (PyC) deposited on SiC fibers (SiC_f_) can improve the mechanical properties of the SiC_f_/SiC composites by preventing fiber/fiber and fiber/matrix integration [5]. Another way based on the layered-structure design of composite can also improve the mechanical properties such as flexural strength and fracture toughness [6,7]. The improved mechanical properties are attributed to a combination of different toughening mechanisms, such as crack deflection, crack bridging, crack branching and delamination, pull-out, and layers rupture.

The development of SiC_f_/SiC ceramic matrix composites (CMCs) is the promising approach to fabricate structural materials for aerospace and nuclear industries [8,9]. Both the SiC matrix and fiber have high hardness values that do not depend on geometric features [10]. This provides for SiC_f_/SiC composites great potential for prospective applications, namely SiC_f_/SiC composites can be used to produce nuclear structural materials with more complex geometry. Due to their superior properties, SiC_f_/SiC composite fuel elements have broad prospects for application in the field of nuclear energy systems and are considered to be ideal candidates for structural materials of nuclear reactors [9,11].

Preceramic paper can be used as a feedstock to fabricate ceramic–based bodies with complex geometry [12,13]. The filler has been incorporated into the preceramic paper in the form of powder. Compared to other sintered ceramic preforms, preceramic paper can be formed into three-dimensional objects by sophisticated paper processing methods. The preceramic paper was used as a feedstock for the fabrication of ceramic structures of complex variable shape and size by laminated object manufacturing (LOM) method [14,15,16].

This work presents the first results on fabrication of paper-derived SiC_f_/SiC_p_ composites by spark plasma sintering (SPS). The processing route presented here consists of orderly stacking of the preceramic paper with the SiC fiber layers followed by spark plasma sintering. The sintering of preceramic papers with fibers by SPS offers time-saving approach to incorporate continuous fibers reinforcing into SiC ceramic matrix and to design a layered structure CMCs avoiding brittle fracture and catastrophic failure of the material. The process is easier and faster than continuously reinforced processing like chemical vapor infiltration (CVI) [17], reactive melt infiltration (RMI) [18,19,20], polymer infiltration and pyrolysis (PIP) [21], etc. Therefore, the aim of this research was to analyze the structure and properties of the paper-derived SiC_f_/SiC_p_ composites obtained using spark plasma sintering.

## 2. Materials and Methods 

### 2.1. Synthesis of SiC_f_/SiC_p_ CMCs

**Figure 1:**
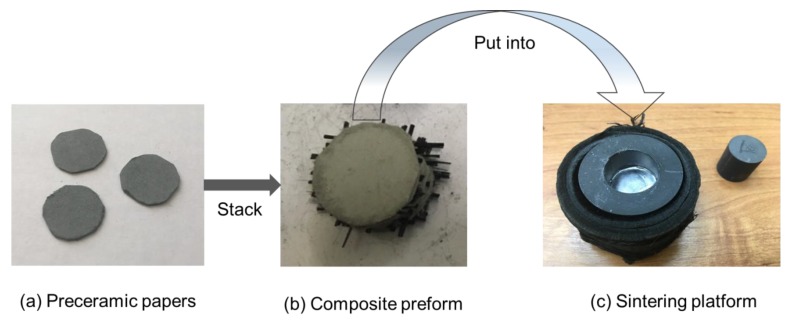
Preparation of preforms for spark plasma sintering: (**a**) cut preceramic paper; (**b**) stacked preceramic paper sheets with fiber layers; (**c**) sintering platform.

A preceramic paper with SiC powder filler was used as a feedstock. The detailed information on fabrication of SiC preceramic paper can be found in Ref. [12]. The steps of preparation of the composite preform before sintering are shown in Figure 1. The preceramic papers were cut into discs of 20 mm in diameter. This size is equal to the inner diameter of the platform used for SPS. 

HI-NICALON™ SiC fibers were used as reinforcing material. These ceramic fibers are uniformly composed of ultrafine β-SiC crystallites and an amorphous mixture of silicon, carbon and oxygen [22,23]. The fiber has excellent strength and modular properties for ceramic fibers and retains its properties at high temperatures. The fiber is highly resistant to oxidation and chemical attack [23]. The fibers with a length of up to 500 m have linear density of about 0.21 g/m.

The cut fibers are placed parallel to the surface of preceramic paper discs. The fibers were laid between the layers of preceramic paper periodically every two layers of paper. The final stack of preceramic paper with SiC fibers is shown in Figure 1b. The aggregation of multiple fibers can reduce the reaction with the SiC matrix [24]. Reinforcement with continuous fiber provides higher strength along the fiber direction but gives poor mechanical properties across the fiber direction [25]. In order to avoid this effect, the angle of 90° is set between two adjacent layers of fibers. The content of fibers was 10 wt.%. The excess fibers in the preform were cut off and the preform was put into a graphite die for sintering. The graphite sheets were placed between the preform and the graphite die and a punch to prevent chemical reactions and ensure good electrical contact during the sintering process (Figure 1c).

The synthesis of the CMCs was carried out using SPS 10–4 machine (GT Advanced Technologies, Santa Rosa, CA, USA). The prepared preform was placed into the 20 mm graphite die between two graphite punches. Maximum sintering temperature was 2100 °C. The sintering time was 3 and 10 min. This is based on preliminary testing to select the lowest feasible temperature and sintering time to minimize the destruction of the fibers during high temperature sintering. The heating rate was 5 °C/s and the cooling rate was 10 °C/s. Pressure was applied until the sample cools down to room temperature to prevent excessive residual stresses in the sample. Considering that the porosity may affect the flexural strength, swelling resistance and other properties of the composites, the sintering pressure was varied from 20 MPa to 60 MPa. To study the effect of the addition of SiC fibers on the mechanical properties of preceramic paper-derived SiC ceramics, the samples without fiber were sintered under the same pressure as the control group. The fiber-free samples were only sintered from stacked preceramic papers by SPS method.

### 2.2. Characterization

After the sintering, the samples were grinded, polished and rinsed with acetone in ultrasonic bath for 15 min. The density of composites was measured by Archimedes’ method using the CP 124S balance machine (Sartorius, Gottingen, Germany). The X-ray computed tomography (CT) was performed using micro-CT scanner TOLMI-150-10 (Tomsk Polytechnic University, Tomsk, Russia) to analyze the macrostructure and defects of the sintered composites. Microstructure and semi-quantitative chemical composition were analyzed by scanning electron microscopy (SEM) using LYRA3 (Tescan, Brno, Czech Republic) and HITACHI^TM^ TM3000 microscopes (Hitachi, Tokyo, Japan) equipped with energy dispersive X-ray (EDX) attachment (Oxford instruments, Abingdon, England). The crystalline structure of the composites was investigated by X-ray diffraction (XRD) analysis using XRD-7000S (Shimadzu, Kyoto, Japan). The scanning parameters: Cu-K_α_ radiation (λ = 0.154 nm), 2θ scan range 10–90°, accelerating voltage 40 kV, current 30 mA, scan speed 10 °/min, sampling step 0.0143°.

### 2.3. Mechanical Testing

**Figure 2:**
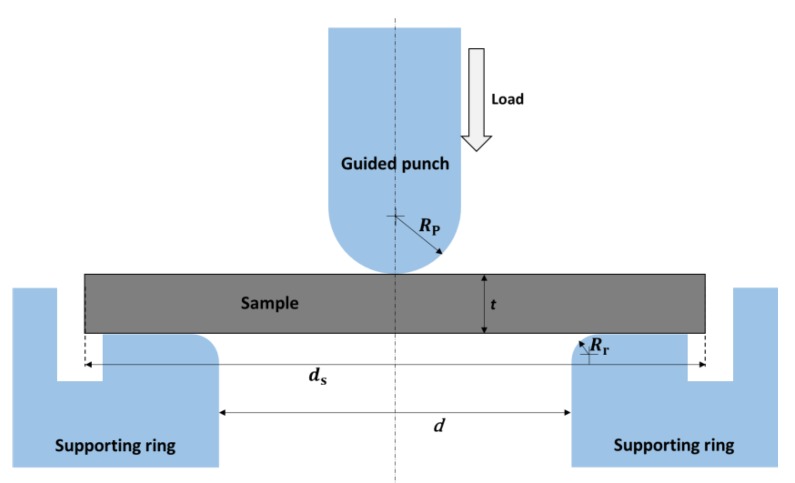
Schematic of the device used for small punch test.

Flexural strength is the most important indicator for evaluating the mechanical properties of ceramic structural materials [26]. Considering that the size of the produced samples is smaller than the minimum size required for the conventional strength test method of brittle materials (not less than 35 mm [27]), and the fibers are distributed in a two-dimensional grid inside the sample, the samples are not suitable for conventional three-point and four-point point flexure tests. Borrowed the design of Manahan et al. [28,29,30,31], in this work a special device is prepared for the small punch (SP) strength test, which is particularly suitable for the testing of very small size samples. The SP test was performed using Al-7000M machine (GOTECH, Taichung, China). Three round pieces of 7.9 mm in diameter were tested for each composite series. The schematic of this device is shown in Figure 2, where *R*_p_ is the radius of the punch tip; *R*_r_ is the radius of the fillet inside the supporting ring; *d* is the inside diameter of the supporting ring; *t* is the thickness of the sample; *d*_s_ is the diameter of the sample. The parameters of the device used for SP test: *R*_p_ = 1 mm, *R*_r_ = 0.5 mm, *d* = 4 mm. In the SP strength test, the disc-shaped sample was supported and centered by a ring and placed centrally by a guided punch with a hemispherical tip. The resulting stress field is axisymmetric. The applied load and displacement of the guided punch are recorded during the test.

The maximum stress at the stressed surface of the sample at the moment of fracture by the SP test is given by empirical equation [32]:

(1) $$\sigma_{\max}=\frac{F}{t^{2}}\left\{\left(1+\nu \right)\left[0.485\ln\left(\frac{R_{d}}{t}\right)+0.52\right]+0.48\right\}$$

where *F* is the fracture load, *t* is the thickness of the disc, *υ* is Poisson’s ratio of the disc material and *R_d_* is the radius of the disc. The substituting parameters of this work were *υ* = 0.2 [33], *ds* = 7.9 mm and *R_d_* = 4 mm. 

## 3. Results and Discussion

### 3.1. Density and Porosity of Sintered Composites

**Figure 3:**
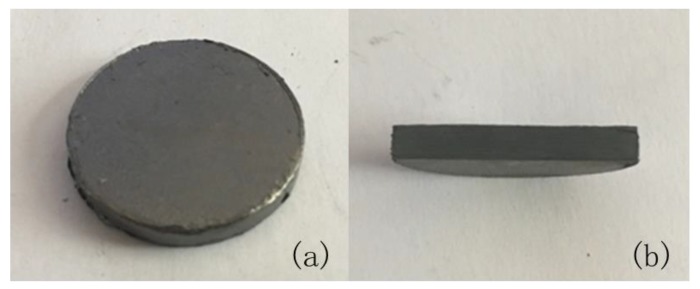
The appearance of the silicon carbide fiber/paper-derived silicon carbide (SiC_f_/SiC_p_) composite sintered at 2100 °C and 40 MPa for 10 min. (**a**) plan view; (**b**) cross section view.

The appearance of the SiC_f_/SiC_p_ composite sintered at 2100 °C and 40 MPa pressure is shown in Figure 3. The composite is a dense pellet with the diameter of 20 mm without visible cracks or delaminations. The surface of the synthesized composite is covered with the graphite layer (100–150 μm), which was subsequently removed by mechanical polishing of the samples. 

During SPS process the organic components (such as cellulose, etc.) decompose and are removed from the preceramic paper, and the remaining SiC matrix is combined and densified with the SiC fibers to form the SiC_f_/SiC_p_ composites. The mass of the composite obtained under different sintering conditions is reduced by about 22% compared to the unsintered preform, which is consistent with the previous results on sintering of preceramic paper-derived SiC ceramics by conventional method [13]. The density, water absorption and porosity for the composites obtained at different sintering conditions are summarized in Table 1.

**Figure 4:**
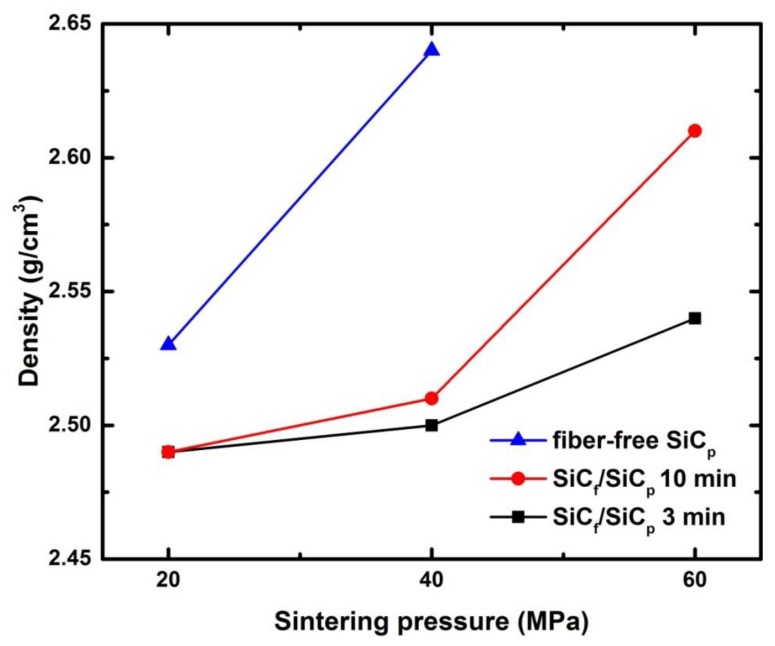
The density of the samples as the function of the sintering pressure.

The density of composites was plotted as the function of the sintering pressure (Figure 4). It can be observed that the addition of SiC fibers results in higher density of the SiC_f_/SiC_p_ composites compared to the fiber-free samples sintered at the same pressures. This evidence will be further confirmed by SEM observations. For each set of different sintering pressures, the density of the samples obtained after sintering for 10 min was higher than that of the samples sintered for 3 min. The density of SiC_f_/SiC_p_ composites increases with increase of the sintering pressure. The highest density of 2.61 g/cm^3^ was achieved for the SiC_f_/SiC_p_ composite sintered under 60 MPa for 10 min. The results on open porosity show that with increase of the sintering pressure, open porosity of composites decreases resulting in lower values of water absorption.

The density of the composites can be expressed by the following formula:

(2) $$r_{f}\times \rho_{f}+\left(1-r_{f}\right)\times \rho_{m}=\rho_{c},$$

where, $\rho_{f}$, $r_{f}$ is the density of the fiber region and its volume fraction after sintering, respectively;$\;\rho_{m}$ is the density of the SiC matrix; $\rho_{c}$ is the density of the SiC_f_/SiC_p_ composite. Considering that the porosity of the fiber-contained region is only ~5% (according to SEM images), we use the density of the fiber region as 0.95 of the density of SiC fibers here. The fibers only account for 10% of the mass fraction of the composite; therefore, the mass fraction is used instead of the volume fraction $r_{f}$ for approximate calculations. The calculated results show that the matrix region densities $\rho_{m}$ of the composites sintered for 3 min with pressure 20 and 40 MPa are 2.51 g/cm^3^ and 2.62 g/cm^3^, respectively. The calculated $\rho_{m}$ is consistent with the measured density of the fiber free sample sintered under the same conditions—2.53 and 2.64 g/cm^3^, respectively (see Table 1). It means that the addition of fibers does not change the density of the SiC matrix formed from the preceramic paper. Only the density of fiber-reinforced layers is strongly affected by the presence of SiC fibers. 

### 3.2. Macro- and Microstructure

**Figure 5:**
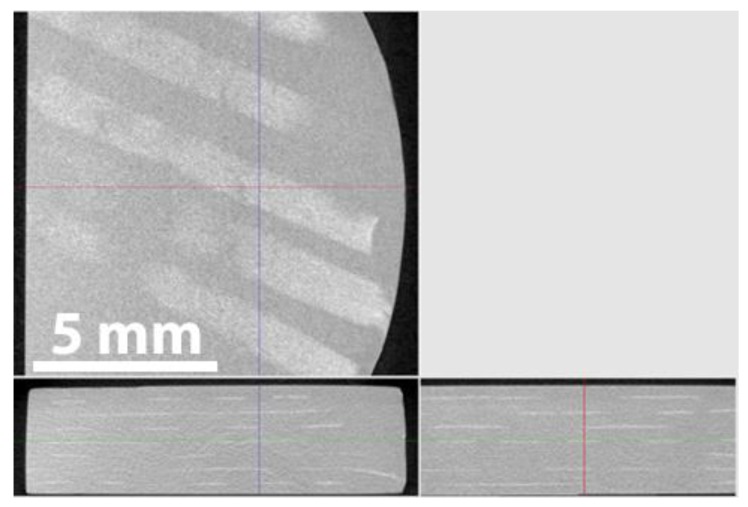
Computed tomography (CT) slices of the SiC_f_/SiC_p_ composites: parallel (**top**) and transverse (**bottom**).

Figure 5 shows the results of X-ray computed tomography (CT) of the SiC_f_/SiC_p_ composites. The reconstructed CT data contains greyscale pictures representing material attenuation of X-rays. The analysis of the parallel and transverse slices showed lighter stripes in the composites, which corresponds to the fiber-containing region. The fiber regions are arranged parallel to the composite surface as expected. The porosity at the resolution of about 6 μm is not observed in all the sintered samples. It has also been found that SiC fibers are retained in the sintered composites.

**Figure 6:**
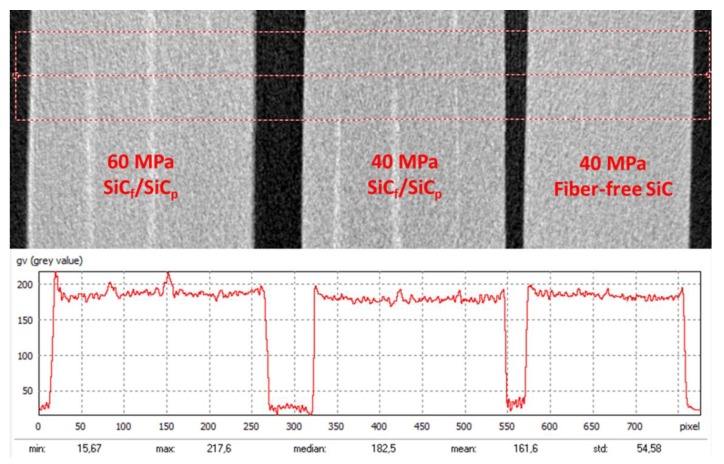
The X-ray density of the samples by the integrated profile.

Comparison of the X-ray density of the samples by the integrated profile show no visible difference between the SiC_f_/SiC_p_ composites sintered at 40 and 60 MPa, except that 60 MPa shows slightly higher average density (Figure 6). The fiber-contained region has higher X-ray density compared to the paper-derived SiC matrix.

**Figure 7:**
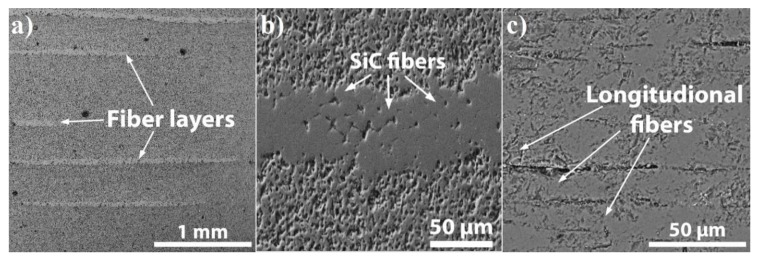
Scanning electron microscopy (SEM) images of SiC_f_/SiC_p_ composites: fiber layers (**a**); cross-section view of SiC fibers (**b**); longitudinal fiber arrangement (**c**).

In order to analyze the microstructure of the composites, the samples were cross-cut and polished, and then observed by scanning electron microscopy. The SEM images of SiC_f_/SiC_p_ composites are shown in Figure 7. It can be seen that the fiber areas (light colored area in Figure 7a) are distributed between the paper-derived SiC matrix layers and contain the aggregation zone of transverse direction fiber (Figure 7b) and longitudinal direction fiber (Figure 7c). It can be clearly observed that the fibers concentrated in each layer are compactly pressed together. Accordingly, the microstructure in the fiber-reinforced layer is denser in comparison with the fiber-free SiC matrix and contains only pores between the fibers.

**Figure 8:**
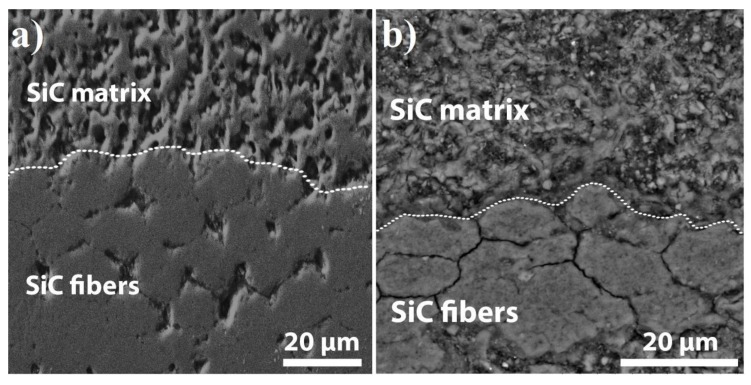
SEM images of SiC_f_/SiC_p_ composites sintered at 60 MPa for 10 min (**a**) and 3 min (**b**).

The magnified cross-section SEM images of the fiber area are shown in Figure 8. The dashed line indicates the fiber contour and the interface between the fiber region and the fiber-free SiC matrix. The partial sintering of the SiC fibers among themselves can be observed in the composite sintered at 60 MPa for 10 min (Figure 8a). Nevertheless, the fibers have an interface and are loosely bonded in the composite obtained by SPS for 3 min (Figure 8b).

**Figure 9:**
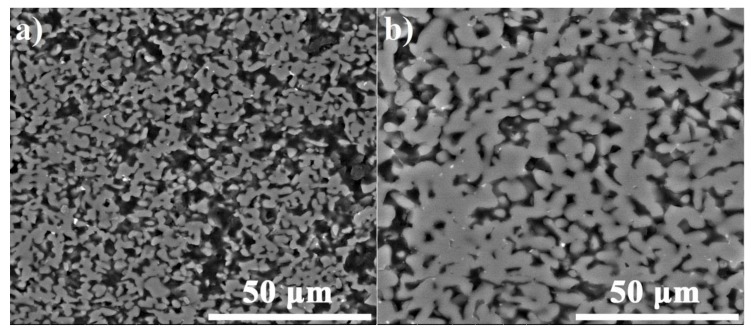
SEM images of the SiC_p_ matrix of the composites sintered at 20 MPa (**a**) and 60 MPa (**b**) for 10 min.

The detailed microstructural observations showed different porosity of the fiber-free SiC matrix depending on the sintering pressure: the higher the sintering pressure is the lower porosity becomes (Figure 9). The pores have a predominantly vertically elongated shape. Due to the application of higher sintering pressure, the SiC particles are much easier fused and compacted in this direction.

### 3.3. Crystalline Structure of The Sintered Composites

**Figure 10:**
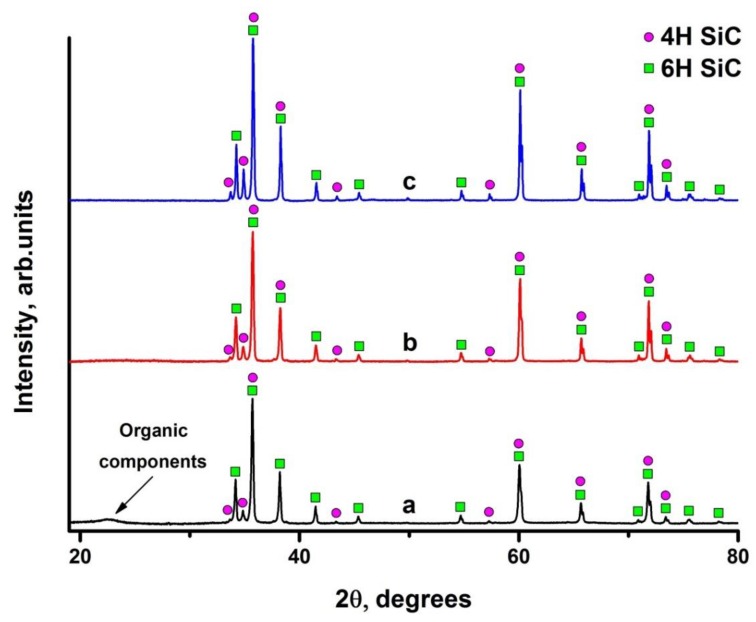
Diffraction patterns of preceramic paper (**a**); preceramic papers-derived SiC (**b**) and SiC_f_/SiC_p_ composite (**c**).

The results of X-ray diffraction analysis of raw material and sintered samples are shown in Figure 10. According to XRD data (Figure 10a), raw SiC preceramic paper consists of two crystalline polymorphic phases (4H and 6H) with hexagonal close package lattice and amorphous phase of organic components (hemicelluloses, cellulose, lignin, pulp fibers, etc.) [12,13]. The volume content of 4H and 6H phases are 15% and 85% respectively. 

The amorphous phase disappears after spark plasma sintering (Figure 10b,c). This is associated with decomposition of hemicelluloses and celluloses at 220–315 °C and 315–400 °C, respectively. At temperature above 400 °C, the charred remains of the pulp fibers start to decompose, and finally they are completely decomposed at 450 °C [26,34]. Lignins decomposes within the wide range of 160–900 °C [35]. This indicates that the organic components in the preceramic papers are completely removed during the sintering process. The phase ratio in the sintered paper-derived SiC_p_ and SiC_f_/SiC_p_ composites slightly changes after SPS process. The content of 4H phase increases to 22 vol.% while content of 6H phase reduces to 78 vol.%. Such distribution between phases is associated with decomposition of organic components during sintering. Obviously, the sintering process has hardly changed the phase composition of the material. The addition of SiC fibers does not change phase composition of the SiC ceramic composites.

### 3.4. Mechanical Properties

The flexural strength was measured to evaluate the mechanical properties of the SiC_f_/SiC_p_ composites. The results of the SP tests for the samples are summarized in Table 2. To analyze the effect of fiber reinforcement, we compared the results for SiC_f_/SiC_p_ composite and fiber-free preceramic paper-derived SiC ceramics sintered at the same pressure (40 MPa) for 10 min. It was shown that the addition of fibers results in enhancement of mechanical properties of the paper-derived SiC ceramics. The SiC_f_/SiC_p_ composite has flexural strength of 360 MPa, which is 20% higher than the fiber-free SiC ceramics (300 MPa). This means that the layered fiber-reinforcement manufacturing process provides the desired effect on mechanical properties of the paper-derived composites. Increase in the sintering pressure improves the mechanical strength of the composites due to formation of denser microstructure.

**Figure 11:**
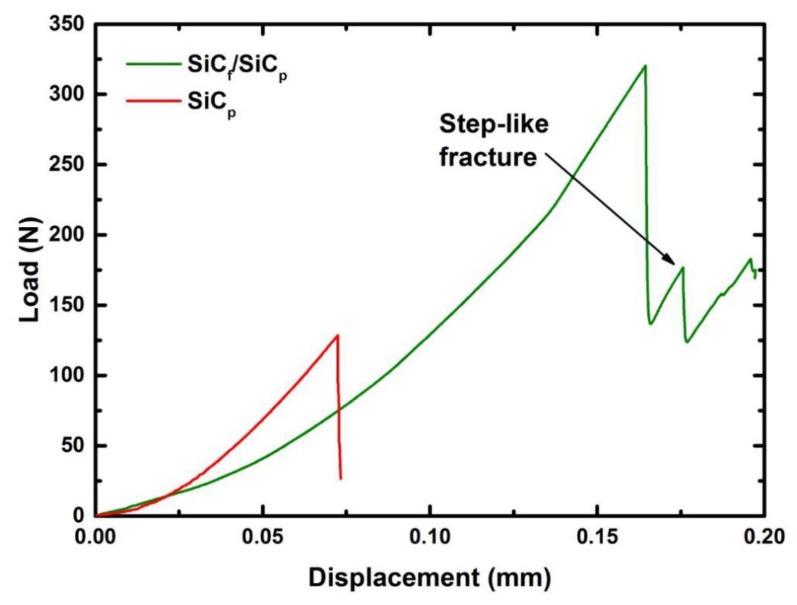
The load–displacement curves of the fiber-free preceramic paper-derived SiC_p_ and SiC_f_/SiC_p_ composite sintered at 40 MPa for 10 min.

The loading-displacement curves of the fiber-free paper-derived SiC and the SiC_f_/SiC_p_ composite are shown in Figure 11. It can be observed that the curve of the fiber-free SiC ceramic indicates brittle fracture of the material. Although the loading curve of the fiber-reinforced composite has similar behavior, the curve did not drop down directly after reaching the highest loading point. The fracture mode in this case is non-catastrophic fracture (step-like fracture), the composite materials exhibit quasi-ductility rather than pure brittleness. The quasi-ductility of the composite is caused by the addition of fibers and layered structure of composite, which is consistent with the results of Nakazato et al. [5] and Nozawa et al. [4].

The maximal flexural strength of 430 MPa was achieved for the composite sintered at 60 MPa for 3 min. Interestingly, the strength of the composite sintered at 60 MPa for 10 min is lower (380 MPa), which is apparently caused by the partial sintering of SiC fibers and, as consequence, led to degradation in their mechanical properties. The maximum strength of 430 MPa meets the strength level of conventional reactive sintered SiC ceramics [36,37], and approximately 80% of the flexural strength of fully dense SiC ceramics (550 MPa). Nevertheless, given the residual porosity in the composite (relatively low density), the strength values are quite high in comparison with the results obtained by other authors [38,39]. The sintering time of 3 min is preferable to synthesize high-strength preceramic paper-derived SiC_f_/SiC_p_ composites by spark plasma sintering method. 

**Figure 12:**
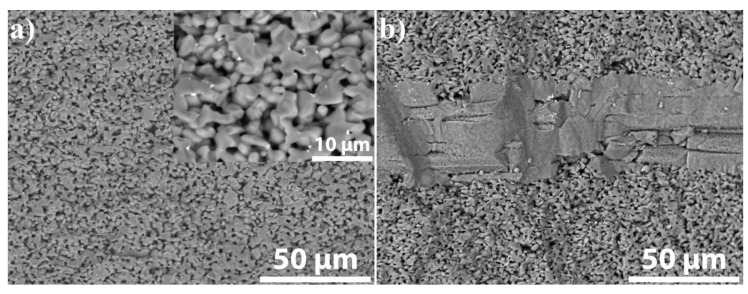
SEM images of the fracture surface of the fiber-free SiC (**a**) with magnified image (inset) and the SiC_f_/SiC_p_ composite sintered at 40 MPa for 10 min (**b**).

The SEM images of the fracture surface of the composites are shown in Figure 12. The fracture surface of the fiber-free SiC ceramic is characterized by a very flush fracture without any obvious local plastic deformation (Figure 12a). Contrarily, the ductile tear ridges formed by local plastic deformation can be observed in the fracture surface of the SiC_f_/SiC_p_ composite (Figure 12b) indicating quasi-ductile fracture mechanism [40].

**Figure 13:**
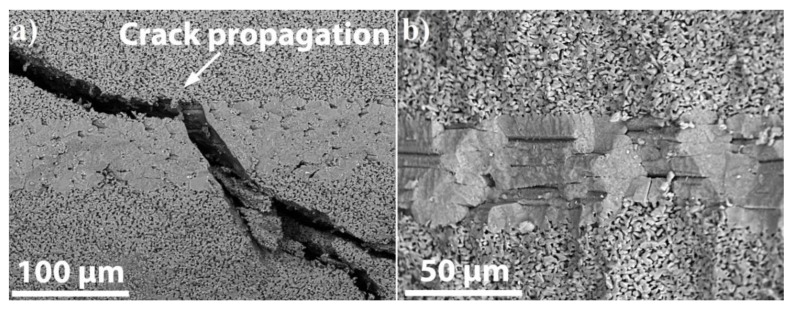
SEM images of fracture surfaces of the SiC_f_/SiC_p_ composite: (**a**) crack propagation in the cross-sectional surface; (**b**) fracture surface.

The stress-induced micro-cracks appeared during the SP bending test are observed on the fracture surface of the SiC_f_/SiC_p_ composite (Figure 13a). During the fracture test, the micro-crack was generated from the top to the bottom. It can be observed that the path of the micro-crack is hindered by the fiber region: the micro-crack is deflected tangential to the interface between matrix and fiber as it approaches the interface, then “refracted” when it enters the fiber region from the matrix. When the micro-crack leaves the fiber region and enters the matrix, it was “refracted” back to the original direction and splits into two cracks. This means that when a micro-crack grows from the matrix to the interface, the crack deviates and splits at the interphase to greatly extend the micro-crack propagation path and dissipate energy. This is consistent with the theory described in the literature of Masuda et al. [41]: the micro-crack initiated from the surface sample propagates to the fiber regions and the crack is arrested by aligned fibers. The micro-crack has to cut through the fibers or the crack propagates between the interfaces. Only partial stretching of fibers between the neighboring fibers was observed (Figure 13b). Thus, the partial sintering of fibers with SiC matrix hindered fiber/matrix detachment. The possible fracture mechanisms responsible for improved flexural strength and stepwise fracture of the SiC_f_/SiC_p_ composite are crack deflection, crack branching etc. Future research will focus on the formation of a protective coating (PyC) to further improve the mechanical properties of such composites due to pull-out effect.

## 4. Conclusions

Novel preceramic paper-derived SiC_f_/SiC_p_ composites were successfully fabricated by the spark plasma sintering method at 2100 °C. The effect of continuous SiC fiber reinforcement as well as of the sintering pressure and time on structure and mechanical properties of the composites was revealed. According to the results, the following conclusions were made:

1. The preceramic SiC-filled papers layer-by-layer reinforced with continuous SiC fibers can be used as raw materials for rapid synthesis of high-strength SiC_f_/SiC_p_ composites.

2. The phase composition of the sintered SiC_f_/SiC_p_ composites corresponds to the composition of the raw pre-ceramic SiC papers. The crystalline structure is represented by α phases 6H-SiC (78%) and 4H-SiC (22%). The organic components of preceramic paper were removed during the sintering process.

3. The SiC fibers are stacked compactly to each other providing the formation of dense reinforced layers between the layers of fiber-free SiC matrix. The latter contributes to the improvement of flexural strength of paper-derived SiC ceramics by approx. 20%. The density of fiber-reinforced composites varies from 2.49 to 2.61 g/cm^3^. The presence of fibers does not change the density of the SiC matrix formed from the preceramic paper.

4. The flexural strength increases with the sintering pressure due to formation of denser microstructure of the SiC matrix. The maximum value of 430 MPa was achieved for the SiC_f_/SiC_p_ composite sintered at 60 MPa for 3 min. Due to partial sintering of the SiC fibers during 10 min sintering the shorter time (3 min) is preferable to obtain high-strength SiC_f_/SiC_p_ composites.

5. The ductile fracture characteristic was observed in the fracture surface of the fiber-reinforced SiC_f_/SiC_p_ composites compared to the pure brittle fracture of the fiber-free paper-derived SiC ceramics. The improved flexural strength and stepwise fracture of the SiC_f_/SiC_p_ composite are attributed to toughening mechanisms such as crack deflection, crack branching, etc.

## Figures and Tables

**Table 1:** Properties of the silicon carbide fiber/paper-derived silicon carbide (SiC_f_/SiC_p_) composite materials sintered at 2100 °C.

Sample #	Sintering Pressure (MPa)	Sintering Time (min)	Fiber	Apparent Density (g/cm^3^)	Water Absorption (%)	Porosity (%)
SiC_f_/SiC_p_-20-3	20	3	yes	2.49	7.0	21.2
SiC_f_/SiC_p_-20-10	20	10	yes	2.49	7.6	21.2
SiC_p_-20-10	20	10	no	2.53	6.5	20.0
SiC_f_/SiC_p_-40-3	40	3	yes	2.50	6.4	20.9
SiC_f_/SiC_p_-40-10	40	10	yes	2.51	6.4	20.7
SiC_p_-40-10	40	10	no	2.64	5.8	16.6
SiC_f_/SiC_p_-60-3	60	3	yes	2.54	6.9	19.6
SiC_f_/SiC_p_-60-10	60	10	yes	2.61	5.8	17.3

**Table 2:** Flexural strength of SiC_f_/SiC_p_ composites and fiber-free SiC_p_ ceramic.

Sample #	Sintering Pressure (MPa)	Sintering Time (min)	Fiber	Bending Strength (MPa)
SiC_p_-40-10	40	10	no	300
SiC_f_/SiC_p_-40-10	40	10	yes	360
SiC_f_/SiC_p_-60-10	60	10	yes	380
SiC_f_/SiC_p_-60-3	60	3	yes	430
